# Intention to Treat Analysis: Are We Really Doing It?: Reply

**DOI:** 10.1007/s00268-013-1984-y

**Published:** 2013-03-14

**Authors:** Marcin Barczyński

**Affiliations:** Third Chair and Department of General Surgery, Jagiellonian University Medical College, 37 Prądnicka Street, 31-202 Kraków, Poland

This reply addresses a response to an article my colleagues and I wrote on the subject of the principle of intention-to-treat (ITT), which has become widely accepted for the analysis of controlled clinical trials [1]. Ideally, in order to preserve fully the immense benefit of randomization, all randomized participants should be included in the analysis, and all should be retained in the group to which they were allocated. However, a strict ITT analysis is often hard to achieve, for two main reasons: missing outcomes for some participants and non-adherence to the trial protocol [2].

Indeed, most randomized trials have some missing observations. A few missing outcomes do not cause a problem, if the power of the trial is not lost by a reduced total sample size, and if loss to follow-up is not related to a patient’s response to treatment. There should be concern when the frequency or the causes of dropout differ between the intervention groups. Participants with missing outcomes can be included in the analysis only if their outcomes are imputed (estimated from other information that was collected). Imputation of the missing data allows the analysis to conform to ITT analysis but requires strong assumptions, which may be hard to justify; otherwise the study should not be undertaken. Finally, ITT corresponds to analyzing the groups exactly as randomized, regardless of adherence to the protocol. One of the noteworthy specific changes in the current CONSORT 2010 Statement was to replace the notion of ITT analysis, a widely misused term, by a more explicit request for information about retaining participants in their original assigned groups. Thus, in the CONSORT checklist, the specific request for ITT analysis has been discarded in favor of a clear description of exactly who was included in each analysis [2].

Mohanty et al. have raised a few questions in their Letter to the Editor about our recently published randomized controlled trial of visualization versus neuromonitoring of the external branch of the superior laryngeal nerve (EBSLN) during thyroidectomy [1]. Those questions merit a brief response. In our study, 210 consenting female patients considered for total thyroidectomy were randomly assigned to two groups equal in size (*n* = 105): visual inspection of the EBSLN and recurrent laryngeal nerve (RLN) versus this visual inspection plus EBSLN and RLN neural monitoring. The primary outcome was the identification rate of the EBSLN. The secondary outcomes included anatomical variability of the EBSLN according to the Cernea classification, and changes in postoperative voice performance during a six-month follow-up. Voice assessment included preoperative and postoperative videostrobolaryngoscopy and an analysis of maximum phonation time (MPT), voice level (VL), fundamental frequency (Fo), and voice quality rating on the GRBAS scale [1]. Males were excluded in order to maintain a homogeneous study group. Of note, 90 % of thyroid surgery is undertaken in women, and many voice parameters evaluated in this study, like MPT, VL, and Fo, differ significantly between men and women. Similarly, patients with a large goiter were not included in this study, also to ensure the homogeneous population of the study group. As the Cernea classification categorizes the nerve in relation to superior thyroid vessels and the upper edge of the superior thyroid pole, the larger the goiter the higher the incidence of type 2B nerve, which was reported in up to 54 % of patients with goiters above 100 ml in volume [3]. Identification of the anatomical variation of the EBSLN in our study was calculated for nerves at risk and not for patients. Significant differences in distribution of all types of the EBSLN according to the Cernea classification between both study groups were the result of improved identification rate of all types of the nerve variants with nerve monitoring system. Nerve stimulation technique has a substantial advantage in identifying all nerve types, including Cernea type 1, which is found at a higher position and sometimes is crowded under the laryngeal head of the sternothyroid muscle, as well as descending types 2A and 2B, which are most vulnerable to surgical manipulation injury. In the study by Selvan et al. [4], a considerable number of false-positive results were seen when visual identification alone was used prior to electrical stimulation of the nerve. In many cases, non-neural fibers or tendinous fibers of the cricothyroid or inferior constrictor muscles were wrongly assumed to be the EBSLN, but were unmasked by the lack of an action potential when stimulated. This finding suggests that visual identification of the EBSLN without electromyographic confirmation may likely be flawed. It is our belief that adding quantitative data to this technique could make the process of nerve identification and preservation more definitive and precise.

To detect a difference of 5 % in the prevalence of primary or secondary outcomes in our trial, which is in agreement with the study of Hurtado-Lopez et al. [5], with a two-sided 5 % significance level and a power of 90 %, a sample size of 105 patients (210 nerves at risk) per group was necessary, given an anticipated dropout rate of 10 %. All the recruited patients received the planned intervention and the identification rate of the EBSLN (primary outcome) and anatomical variability of the EBSLN according to the Cernea classification (one of the secondary outcomes) were analyzed in all 210 patients included in the study, which is clearly reported in the Results section and summarized in our Table 2. However, 4 (3.8 %) patients operated on without nerve monitoring versus 5 (4.7 %) patients operated on with nerve monitoring were lost to follow-up (non-significant difference; 5 patients opted to withdraw from the trial, 2 patients moved away, and 2 patients became unavailable for unknown reasons), whereas the remaining 201 patients completed the six-month follow-up [1]. Thus, the actual dropout rate for evaluation of changes in postoperative voice performance (the other secondary outcome) was far below the anticipated dropout rate of 10 %, and the power of the trial has not been lost by this reduction in number of the analyzed participants. Any imputation of the missing postoperative voice performance data was not done, as they were impossible to be reliably estimated, and it was also not necessary from the statistical point of view, because the study was not in danger of being underpowered. In addition, the outcomes of this study were analyzed in the subgroups exactly as randomized. Taking into consideration all the above points, I must strongly disagree with the suggestion raised by Mohanty et al. in their letter that the analysis of data in our trial was per protocol rather than ITT.
